# The effects of orally administered lactoferrin in the prevention and management of viral infections: A systematic review

**DOI:** 10.1002/rmv.2261

**Published:** 2021-05-28

**Authors:** Alessandra Sinopoli, Claudia Isonne, Maria Mercedes Santoro, Valentina Baccolini

**Affiliations:** ^1^ Department of Prevention Local Health Unit Roma 1 Rome Italy; ^2^ Department of Experimental Medicine University of Rome “Tor Vergata” Rome Italy; ^3^ Department of Public Health and Infectious Diseases Sapienza University of Rome Rome Italy

**Keywords:** COVID‐19, lactoferrin, SARS‐CoV‐2, systematic review, viral infections

## Abstract

It has been demonstrated that lactoferrin (LF) plays a role in host defence, but evidence on its potential antiviral property from clinical studies is fragmented. Our systematic review aimed at identifying the effects of orally administered LF against virus infections. The systematic search was conducted on PubMed, Scopus, Web of Science, BioRxiv.org and ClinicalTrials.gov from database inception to 7th January 2021. Eligible articles investigated any virus family and provided data on the effects of orally administered LF of any origin in the prevention and/or management of confirmed viral infections in people of any age. A narrative synthesis of the results was performed. Quality was assessed with the Cochrane Risk‐Of‐Bias and ROBINS‐1 tools. A total of 27 records were included, nine of which were registered protocols. We found data on *Flaviviridae* (*n* = 10), *Retroviridae* (*n* = 3), *Coronaviridae* (*n* = 2), *Reoviridae* (*n* = 2) and *Caliciviridae* (*n* = 1). Most published trials were at high risk of bias. The findings were heterogeneous across and within viral families regarding virological, immunological and biological response, with no clear conclusion. Some weak but positive results were reported about decrease of symptom severity and duration, or reduction in viral loads. Despite high tolerability, the effects of LF as oral supplement are still inconsistent, both in preventing and managing viral infections. Small sample sizes, variety in recruitment and treatment protocols, and low study quality may have contributed to such heterogeneity. Better‐designed studies are needed to further investigate its potential benefits against viral infections, including SARS‐CoV‐2.

**Abbreviations**
bLF: bovine lactoferrin
COVID: coronavirus disease
hLF: human lactoferrin
LF: lactoferrin
SARS‐CoV‐2: coronavirus virus 2

## 1 INTRODUCTION

Lactoferrin (LF) is a multifunctional glycoprotein, member of the transferrin family,^1^ identified for the first time in 1939 in bovine milk and isolated in 1960 from human milk.^2^, ^3^ Several studies have demonstrated that it plays a role in host defence^4^, ^5^; because of its structure, it is a component of the innate immune response and a potent immunomodulator.^6^ Its ability to bind free iron ions^7^ prevents the tissues from excessive inflammatory processes.^8^ Additionally, it is now widely recognized for antioxidant activity^9^, ^10^ and antibacterial activity,^11^ and Bezault et al. presented convincing data about its anti‐cancer activity in murine models of fibrosarcoma and melanoma.^12^ In the 1980s, some authors documented for the first time that LF may also affect the myelopoiesis of mice inoculated with a friend virus complex,^13^ paving the way for other hypotheses about the role of LF in viral infections.^14^

To date, the antiviral property of LF has been confirmed by several in vitro studies.^15^, ^16^, ^17^ It is directed against a broad spectrum of viruses, including both RNA‐ and DNA‐viruses, enveloped as well as naked viruses.^15^ Some studies have indicated that LF prevents infection of the host cell, rather than inhibiting virus replication in the target cell,^18^, ^19^ whereas other authors have demonstrated its ability to prevent viral infections by acting through interaction with heparan sulfate proteoglycans^20^ in a dose‐dependent effect,^21^ by binding to viral particles or viral receptors, and by involving apoptosis or inflammatory pathways.^15^ Another mechanism of action is the upregulation of the antiviral response of the immune system.^22^, ^23^ In fact, NK‐cells, monocyte/macrophages and granulocytes play an essential role during the early phases of a viral infection,^24^ and polymorphonuclear leukocytes seem to become more effective after exposure to LF, thanks to greater motility and faster production of superoxide.^25^, ^26^

Over the last year, the severe acute respiratory syndrome coronavirus virus 2 (SARS‐CoV‐2) has captured the attention of the scientific community. With the rapidly escalating situation worldwide, researchers have sought treatment strategies to control this infection.^26^, ^27^ As vitamin and mineral insufficiency was observed in COVID‐19 patients at increased risk of mortality,^28^ dietary supplements and drugs have been considered as supportive therapy.^29^ Relying on some positive results,^23^, ^30^ some authors proposed LF as a supplemental treatment for COVID‐19,^31^ but evidence on its potential effects from clinical studies is still fragmented. The aim of our systematic review was to identify the effects of orally administered LF against viral infections, with a specific focus on those caused by coronaviruses, to provide a synthesis of the results and support clinicians in the evaluation of supplemental treatments for COVID‐19.

## 2 METHODS

This study was conducted according to the Cochrane Handbook for systematic reviews and the Preferred Reporting Items for Systematic Reviews and Meta‐Analyses statement.^32^, ^33^

### 2.1 Inclusion criteria

Eligible articles had any primary study design, were conducted in any country, reported in English or Italian, investigated any virus family and provided data on the effect of orally administered LF of any origin (e.g., bovine [bLF], human [hLF] or others) in the prevention and/or management of confirmed viral infections in people of any age. No minimum LF dosage was specified. Articles using in vitro techniques, conducted on animals, exploring the relationship between LF and bacteria, fungi, parasites or unspecified microorganisms, or focussing only on the glycoprotein's capacity to stimulate the participants' immune response without a confirmed viral infection were excluded.

### 2.2 Search strategy

To reach adequate coverage of the clinical research conducted on the topic, two reviewers independently searched PubMed, Scopus and Web of Science from database inception to 7th January 2021 using the following terms: lactoferrin AND covid OR mers OR sars OR coronavirus OR virus OR hcov OR hku1. The string was adapted to fit the search criteria of each database (Table S1). No filter was applied in the search strategy. Duplicate articles were removed, and the title and abstract of the collected records were screened. Studies that clearly did not meet the inclusion criteria were excluded. Full texts of potentially relevant articles were retrieved and independently examined by two researchers. Disagreements were resolved through discussion, and reasons for exclusion recorded. The reference lists of retrieved articles were also searched to identify other potentially relevant studies.

Additionally, BioRxiv.org was searched as a pre‐print database using the string ‘lactoferrin AND virus’ and ‘lactoferrin AND SARS‐CoV‐2’ whereas ClinicalTrials.gov was queried to map planned, ongoing or just completed clinical studies. The same screening process mentioned for the bibliographic databases was applied.

### 2.3 Data collection and synthesis

For each eligible study retrieved from the literature search (i.e., published or pre‐print), two reviewers independently extracted the following information: first author, year of publication/submission, country, virus family, characteristics of the target population, study design, type and duration of the intervention, area of evaluation (prevention or management of viral infections), main findings and side effects. Articles were grouped according to the virus family, and a narrative synthesis was performed. As for the records investigating the LF effect on the management of viral infections, three categories were considered: virological response, immunological response and biological response. Two independent authors performed the quality assessment of the articles included in the systematic review using the revised Cochrane Risk‐Of‐Bias tool version 2^34^ for randomized studies and the ROBINS‐I tool^35^ for non‐randomized interventions. Discrepancies were resolved by consensus or by a third reviewer. Judgements on the quality of the studies followed the Cochrane guidelines.^34^, ^35^

For each record retrieved from ClinicalTrials.gov, two reviewers independently extracted the following information: principal investigator and identifier, title, country, start and completion date, condition or virus being studied, purpose/outcome, recruitment status. A narrative synthesis of the results was performed.

## 3 RESULTS

**FIGURE 1:**
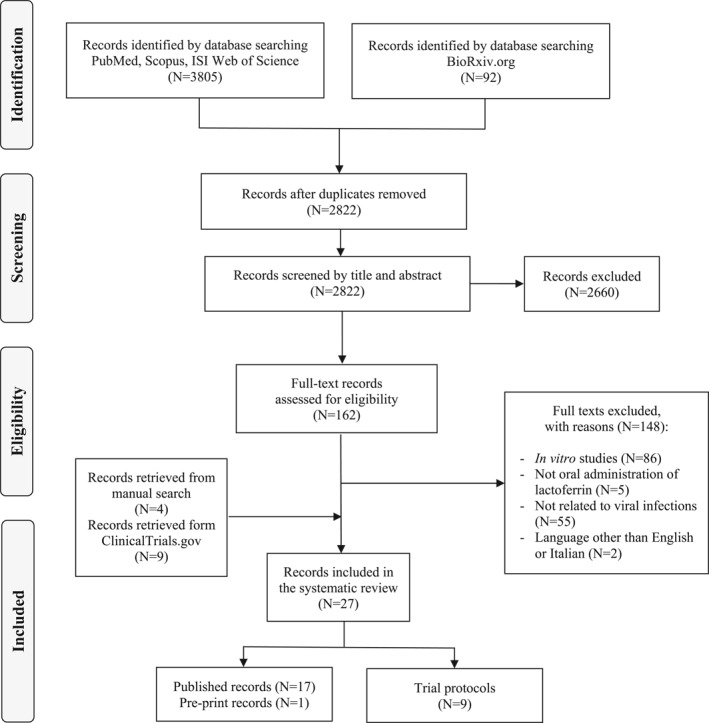
PRISMA flow diagram of the review process

After the removal of duplicates, 2822 records resulted from the initial search (Figure 1). Screening by title and abstract selected 162 articles eligible for full‐text analysis, from which 148 records were excluded with reasons. Four records were added to the previous 14 from the reference lists of relevant articles, and nine records retrieved from ClinicalTrials.gov met our inclusion criteria, for a total of 27 records included in the systematic review.

### 3.1 Literature search

#### 3.1.1 Characteristics of the included studies

We found data on infections sustained by hepatitis C virus (HCV; *n* = 10, *Flaviviridae*)*,* human immunodeficiency virus (HIV; *n* = 3, *Retroviridae*), SARS‐CoV‐2 (*n* = 2, *Coronaviridae*), Rotavirus (*n* = 2, *Reoviridae*) and Norovirus (*n* = 1, *Caliciviridae*) (Table 1). Studies were published between 1999 and 2020 and carried out mostly in Japan (*n* = 10), followed by Italy (*n* = 3), Egypt (*n* = 1), United States (*n* = 1), Taiwan (*n* = 1), Spain (*n* = 1) and Peru (*n* = 1). There was considerable heterogeneity in the recruitment protocols and interventions. Nine were randomized trials,^36^, ^37^, ^38^, ^39^, ^40^, ^41^, ^42^, ^43^, ^44^ eight did not report a random allocation of patients,^45^, ^46^, ^47^, ^48^, ^49^, ^50^, ^51^, ^52^ and one study investigated one group only.^53^ Treatment duration ranged from 10 days^51^ to 15 months.^37^ The studies predominantly enrolled a population aged >18 years, whereas five articles considered children.^43^, ^44^, ^49^, ^50^, ^53^ Among studies targeting adult individuals, 10 were on patients with chronic hepatitis C,^36^, ^37^, ^38^, ^39^, ^40^, ^41^, ^45^, ^46^, ^47^, ^48^ one enrolled adults infected with HIV^42^ and two studies examined patients infected with SARS‐CoV‐2.^51^, ^52^ Among articles studying a paediatric population, two evaluated HIV infected children^49^, ^53^ and three considered healthy participants.^43^, ^44^, ^50^ As for the quality assessment, all but one^39^ randomized trial were judged at high risk of bias (*n* = 6)^36^, ^37^, ^38^, ^40^, ^41^, ^43^ or with some concerns (*n* = 2)^42^, ^44^: main deficits were found in the risk of bias arising from the randomization process and the risk of bias due to deviations from the intended interventions (Table S2). Similarly, the overall bias in non‐randomized studies was deemed as critical^47^, ^50^, ^51^ or serious^45^, ^49^, ^52^, ^53^ in most cases (*n* = 3 and *n* = 4, respectively), and moderate in the remaining two studies^36^, ^46^ (Table S3).

**TABLE 1:** Characteristics of the studies retrieved from the literature search and included in the systematic review

Virus family	Virus	First author, year	Country	Study design	Treatment duration	Study population	Risk of bias or overall bias
*Flaviviridae*	HCV	Tanaka, 1999	Japan	NRS	2 months	11 patients with CHC	Critical
	HCV	Iwasa, 2001	Japan	R‐DRT	6 months	25 patients with CHC	High
	HCV	Okada, 2002	Japan	NRS	2 months	45 patients with CHC	Moderate
	HCV	Ishii, 2003	Japan	RCT	12 months	63 patients with CHC	High
	HCV	Hirashima, 2004	Japan	RCT	50 weeks	21 patients with CHC	High
	HCV	Ishibashi, 2005	Japan	RCT	6 months	36 patients with CHC	High
	HCV	Kaito, 2006	Japan	RCT	2 months	111 patients with CHC	High
	HCV	Konishi, 2006	Japan	NRS	2 months	90 patients with CHC	Moderate
	HCV	Ueno, 2006	Japan	RCT	3 months	198 patients with CHC	Low
	HCV	El‐Ansary, 2017	Egypt	NRS	3 months	60 patients with CHC	Serious
*Caliciviridae*	Norovirus	Ochoa, 2013	Peru	RCT	6 months	555 healthy children	Some concerns
*Coronaviridae*	SARS‐CoV‐2	Serrano, 2020	Spain	NRS	10 days	75 patients with COVID‐19	Critical
	SARS‐CoV‐2	Campione, 2020^a^	Italy	NRS	1 month	92 patients with COVID‐19	Serious
*Retroviridae*	HIV	Zuccotti, 2006	Italy	NRS	6 months	22 children with HIV	Serious
	HIV	Zuccotti, 2007	Italy	NRS	1 month	11 children with HIV	Serious
	HIV	Sortino, 2019	United States	RCT	3 months	54 patients with HIV	Some concerns
*Reoviridae*	Rotavirus	Egashira, 2006	Japan	NRS	12 weeks	298 healthy children aged <5 years	Critical
	Rotavirus	Yen, 2010	Taiwan	RCT	15 months	216 healthy children aged 2–6 years	High

Abbreviations: CHC, chronic hepatitis C; COVID, coronavirus disease; HCV, hepatitis C virus; HIV, human immunodeficiency virus; NRS, non‐randomized study; R‐DRT, randomized dose‐response trial; RCT, randomized controlled trial; SARS‐CoV‐2, severe acute respiratory syndrome coronavirus virus 2.

^a^ Pre‐print article.

#### 3.1.2 Flaviviridae

Three studies compared a different daily dosage of bLF,^38^, ^46^, ^47^ three studies compared bLF to no therapy,^37^, ^39^, ^48^ two studies compared a combination of bLF and IFN‐*α* or a combination of bLF, IFN‐*α* and ribavirin to the same therapeutic regimen without bLF,^40^, ^41^ one study compared bLF directly to ribavirin plus IFN‐*α* ^45^ and one study both bLF versus placebo as well as bLF‐ribavirin‐IFN‐*α* triple therapy and ribavirin‐IFN‐*α* therapy.^36^ The bLF dose varied consistently across the studies, from a minimum of 0.4 g/day^38^ to a maximum of 7.2 g/day^46^ (Table 2).

**TABLE 2:** Main effects of lactoferrin oral administration in the management of patients affected by chronic hepatitis C

Author, year	Treatment	Management			Side effects
		Virological reponse	Immunological response	Biological response	
Tanaka, 1999	Group I: 1.8 g bLF/day; Group II: 6 g bLF/day	Significant HCV‐RNA decrease in patients with low pre‐treatment viral load	NA	Significant decrease in ALT levels in patients with low pre‐treatment viral load	None
Iwasa, 2002	Group I: 0.4 g bLF/day; Group II: 3.6 g bLF/day	Significant HCV‐RNA decrease in the second group	NA	No change in ALT level in both groups	None
Okada, 2002	Group I: 1.8 g bLF/day; Group II: 3.6 g bLF/day; Group III: 7.2 g bLF/day	Non‐significant dose‐response effect	NA	No significant dose‐response effect with ALT level	Minor and dose‐dependent
Ishii, 2003	Group I: 0.6 g bLF/day; Group II: no therapy	Non‐significant difference	Significant increase in IL‐18 level non‐significant difference in IL‐4 and IFN‐*γ* levels	Non‐significant difference in ALT level	None
Hirashima, 2004	Group I: CIFN + 9.0 g bLF/dayGroup II: CIFN	Non‐significant difference	NA	Non‐significant difference in ALT level	Non‐significant difference
Ishibashi, 2005	Group I: IFN‐*α*‐2b + ribavirin + 0.6 g bLF/day; Group II: IFN‐*α*‐2b + ribavirin	Non‐significant difference	NA	Non‐significant difference in ALT level	Non‐significant difference
Kaito, 2006	Group I: 3.6 g bLF/day Group II: no therapy Group I: IFN‐*α* + ribavirin + 3.6 g bLF/day; Group II: IFN‐*α*+ ribavirin	Significant HCV‐RNA decrease significant HCV‐RNA decrease in bLF‐responders	NA	NA	Non‐significant difference when compared to the therapy group
Konishi, 2006	Group I: 3.6 g bLF/day; Group II: no therapy	Non‐significant difference	NA	Significant decrease in ALT level significant decrease in plasma 8‐isoprostane	NA
Ueno, 2006	Group I: 1.8 g bLF/day; Group II: no therapy	Non‐significant difference	Non‐significant difference in IL‐18 level	Non‐significant difference in ALT level	Non‐significant difference
El‐Ansary, 2017	Group I: 0.5 g bLF/day; Group II: IFN‐*α* + ribavirin	Non‐significant difference	Significant higher CD4, CD8, CD137 and CD56 levels	NA	NA

Abbreviations: ALT, alanine transaminase; bLF, bovine Lactoferrin; CIFN, Consensus interferon; IL, Interleukin; NA, not assessed.

All studies analysed the virological response after treatment in terms of HCV‐RNA, with heterogeneous results: one study^38^ found a significant decrease in the viral load after a higher bLF dosage, whereas one study^46^ reported a non‐significant dose‐response effect. Tanaka and colleagues^47^ described a reduction in the outcome only for patients with low pre‐treatment viral load. Most studies reported a non‐significant difference in the virological response between bLF and placebo,^37^, ^39^, ^48^ as well as comparing the additional effect of bLF on a drug therapy^40^, ^41^ or considering bLF in direct comparison to ribavirin plus IFN‐*α*.^45^ Only one study reported a significant decrease in the viral load after bLF in monotherapy or in triple therapy, but among patients classified as bLF‐responders.^36^

The immunological response was investigated in three studies,^37^, ^39^, ^45^ with no consistent results on IL‐18 levels.^37^, ^39^ Oral administration of bLF did not seem to influence IL‐4 and IFN‐*γ* levels,^37^ whereas *El‐Ansary* and colleagues^45^ found significantly higher expression of CD4, CD8, CD137 and CD56 in the bLF group compared to ribavirin plus IFN‐*α*.

Eight studies reported the effects on biological response, mainly in terms of ALT levels. No change was observed according to the bLF dosage,^38^, ^46^ or in general by most studies.^37^, ^39^, ^40^, ^41^ Only one study reported a significant decrease in ALT level^48^ and plasma 8‐isoprostane levels compared to no therapy, while one study found a decrease in ALT serum concentration only in patients with low pre‐treatment viral load.^47^

All studies but two^45^, ^48^ provided data on side effects. Three studies reported none,^37^, ^38^, ^47^ in one study the signs and symptoms reported were minor but dose‐dependent,^46^ whereas in the last four studies the authors did not find any difference between the side effects shown by the patient groups.^36^, ^39^, ^40^, ^41^

#### 3.1.3 Caliciviridae

The only study on calicivirus compared the oral administration of bLF at a dosage of 0.5 g/day to placebo^44^ (Table 3). The potential role of bLF in preventing episodes of diarrhoea in children was the main outcome, but no reduction in diarrhoea incidence was reported. Rather, a decrease in duration and severity of gastroenteritis‐related symptoms was observed.

**TABLE 3:** Main effects of lactoferrin oral administration in the prevention and management of viral infections

Author, year	Treatment	Prevention	Management						Side effects
			Virological Response	Immunological response		Symptom assessment		Other	
*Caliciviridae*									
Ochoa, 2013	Group I: 0.5 g bLF/day; Group II: placebo	No difference in diarrhoea incidence	NA	NA		Decrease in duration and severity of symptoms		NA	NA
*Coronaviridae*									
Serrano, 2020	Group I: 20–30 mg bLF/day + zinc; Group II: 20–30 mg bLF/day	NA	NA	NA		Improvement in the main symptoms in both groups		NA	None
Campione, 2020 (pre‐print)	Group I: 1 g bLF/day; Group II: standard of care treatment; Group III: No therapy control group: Healthy volunteers	NA	Significant decrease in median time length of rRT‐PCR SARS‐CoV‐2 RNA negative conversion (Group I vs. Group II, and Group I vs. Group III)	Significant decrease in IL‐6 and D‐Dimer levels in Group I; non‐significant decrease in TNF‐*⍺* in Group I non‐significant increase in IL‐10 level in Group I non‐significant difference in adrenomedullin level in group I		Significant decrease in duration of symptoms (Group I vs. Group II, and Group I vs. Group III)		Significant decrease in ferritin level in Group I non‐significant difference in serum iron and transferrin levels in Group I	Minor
*Retroviridae*									
Zuccotti, 2006	Group I: 3 g bLF/day without ARV therapy; Group II: 3 g bLF/day + RTI based therapy^a^; Group II: 3 g bLF/day + HAART therapy^b^	NA	Significant decline in viral load during the bLF administration in groups I and II, but non‐significant comparing the two groups	Significant increase in CD4+ cell percentage during the bLF administration in group I and II, but significantly higher in Group II; non‐significant difference in absolute CD4+ cell count in any group		None		NA	None
Zuccotti, 2007	Group I: 3 g bLF/day	NA	No change in viral load	Skewing of T‐lymphocytes towards more differentiated subpopulations; no significant change in absolute CD4+ and CD8+ cell count; Improvement in phagocytosis, killing, TLR‐2 expression and IL‐12/IL‐10 ratio		NA		NA	NA
Sortino, 2019	Group I: 1.5 g rh‐LF/day; Group II: placebo	NA	NA	Non‐significant difference in inflammatory or immunologic outcomes		Non‐significant difference in HIV related symptoms		Significant increase in transferrin saturation; non‐significant difference in intestinal microbiotical effects	Non‐significant difference
*Reoviridae*									
Egashira, 2007	Group I: 100 mg bLF/day; Group II: Placebo	Non‐significant difference in gastroenteritis' incidence	NA		NA	Significant decrease in the frequency and duration of symptoms	NA		NA
Yen, 2011	Group I: 70–80 mg bLF/day; Group II: placebo	Non‐significant difference in gastroenteritis' incidence	NA		Non‐significant difference in the IFN‐gamma and IL‐10 levels between two groups	Non‐significant difference	NA		NA

Abbreviations: ARV, antiretroviral; bLF, bovine lactoferrin; HAART, highly active antiretroviral therapy; IL, interleukin; NA, not assessed; NRTI, nucleoside reverse‐transcriptase inhibitor; RTIs, reverse‐transcriptase inhibitor; rh‐LF, recombinant human lactoferrin.

^a^ Antiretroviral therapy based on two NRTIs or one NRTI plus one non‐NNRTI.

^b^ Triple antiretroviral therapy regimen including two NRTIs and one protease inhibitor.

#### 3.1.4 Coronaviridae

One of the two studies that enrolled patients with COVID‐19 is still in the pre‐print version^52^ (Table 3). The bLF daily dose varied from 20–30 mg to 1 g. One study^51^ reported symptoms improvement only and no side effects. The other^52^ compared bLF to different treatment groups (standard of care, no therapy and healthy volunteers), finding a significant decrease in the median time length of rRT‐PCR SARS‐CoV‐2 RNA negative conversion both between bLF and standard of care and between bLF and no therapy. A few immunological outcomes improved in the bLF‐supplemented group (IL‐6, D‐Dimer), but others did not (TNF‐*⍺*, Il‐10, adrenomedullin). By contrast, a significant decrease in the duration of symptoms was observed coupled with a reduction in ferritin levels, but no changes in iron and transferrin levels were found. Minor side effects were mentioned.^52^

#### 3.1.5 Retroviridae

Two studies used the same daily dosage of bLF^49^, ^53^ while one study compared 1.5 g daily administration of recombinant hLF to placebo^42^ (Table 3). None of them investigated preventive effects. The virological response was assessed in two studies,^49^, ^53^ and only one^49^ found a significant decrease in the viral load during bLF administration in patients that received no antiretroviral therapy (group I) or considering those who received a combination of two antiretroviral agents (Group II), with no difference between the two groups. The immunological response was evaluated in all three studies but heterogeneously. A differentiation of subpopulation T‐lymphocytes and an improvement in phagocytosis and killing, Toll‐like receptor expression, and IL‐12/IL‐10 ratio were found in one study^53^; CD8+ cell count was assessed once, with no meaningful findings^49^; absolute CD4+ cell count seemed to not improve in two studies,^49^, ^53^ whereas one study^49^ found an increase in the CD4+ cell percentage in groups I and II during bLF administration, even though it was higher for the latter. The study that compared rh‐LF and the placebo group did not highlight a significant difference in immunological response.^42^

Symptoms related to the underlying disease were evaluated in two studies^42^, ^53^ without significant results. The most recent paper also studied intestinal microbiological effects, reporting no significant differences between the two groups but an increase in transferrin saturation.^42^ Lastly, side effects were found to not differ between the two groups in one study.^42^

#### 3.1.6 Reoviridae

Reoviridae were analysed in two studies that compared the effects of bLF administration at a daily dosage of 70–80 mg^43^ and 100 mg^50^ versus placebo (Table 3). No significant differences were found between the two groups in preventing gastroenteritis onset in both trials, whereas they yielded heterogeneous results for the assessment of the symptoms. Also, the immunological response was investigated in one study only,^43^ with no clinically meaningful findings.

### 3.2 ClinicalTrials.gov

The nine records retrieved from ClinicalTrials.gov were randomized trial protocols (Table 4). Three were registered in Egypt, two in the United States, two in Peru, one in Pakistan and one in Italy. Four are reported as completed, but only three have published their results and the respective full‐text articles are included in the systematic review.^42^, ^44^, ^52^ One trial investigating the immunological response after poliovirus vaccination in children is still recruiting participants. The remaining four protocols focus on SARS‐CoV‐2 but are reported as not yet recruiting.

**TABLE 4:** Records retrieved from Clinicaltrials.gov investigating the effects of orally administered LF in the prevention and/or management of viral infections

Principal investigator, identifier	Title	Country	Start date, Completion date	Condition or disease, virus	Purpose/Outcome	Recruitment status
Cleary, NCT00560222	Randomized, controlled trial—LF prevention of diarrhoea in children	Perù	Feb 2008, Oct 2011	Diarrhoea and malnutrition	To determinate the effect of bLF on diarrhoea prevention and on growth in intervention group of healthy children versus placebo group	Completed and published^44^
Allen, NCT01092039	XIGO effectiveness study: An investigation of the safety and efficacy of oral XIGO tablets on patients diagnosed with the common cold	United States	Mar 2010, Apr 2011	Common cold	To assess symptoms severity and resolution after bLF administration in intervention group of patients with cold versus placebo group	Completed
Baker, NCT01830595	Recombinant LF to reduce immune activation and coagulation among HIV positive patients	United States	Sep 2014, Jan 2018	HIV	To evaluate effectiveness of bLF in reducing inflammation (IL‐6, D‐dimer, CD‐16, sCD163) in intervention group of HIV infected patients versus placebo group	Completed and published^42^
Campione,NCT04475120	Interventional pilot study to assess the use of oral and intra‐nasal liposomal LF in COVID‐19 patients with mild‐to‐moderate disease and in COVID‐19 asymptomatic patients	Italy	Apr 2020, Jul 2020	SARS‐CoV‐2	To assess the efficacy of liposomal LF in COVID‐19 patients with mild‐to moderate disease and in COVID‐19 asymptomatic patients versus no intervention groups	Completed and published^52^ ^,^ ^a^
Soofi, NCT04432935	Effect of bovine LF on seroconversion following polio vaccine administration in children: A randomized control trial	Pakistan	Jun 2020, Sep 2021 (estimated)	Poliomyelitis	To evaluate the level of seroconversion in children following poliovirus vaccination after bLF administration versus placebo group	Recruiting
Esmat, NCT04421534	Utility of LF as an adjunct therapeutic agent for COVID‐19	Egypt	Jun 2020, Sep 2020	SARS‐CoV‐2	To study the potential application of LF against SARS‐CoV‐2 and propose the possibility of using different doses of supplemental LF as a potential adjunct treatment for COVID‐19	Not yet recruiting
Hegazy, NCT04412395	Clinical assessment of oral LF as a safe antiviral and immunoregulatory in treating COVID‐19 disease (COVID‐19_LF)	Egypt	Jun 2020,Sep 2021 (estimated)	SARS‐CoV‐2	To clinically use bLF as a safe antiviral adjuvant for treatment and to assess the potential in reducing mortality and morbidity rates in COVID‐19 patients	Not yet recruiting
Esmat, NCT04427865	Utility of LF as a preventive agent for healthcare workers exposed to COVID‐19	Egypt	Jul 2020, Nov 2020	SARS‐CoV‐2	To assess the safety and efficacy of LF within the context of SARS‐CoV‐2 and propose the possibility of supplemental LF as a potential preventive drug for healthcare workers exposed to SARS‐CoV‐2	Not yet recruiting
Ochoa, NCT04526821	LF for prevention of COVID‐19 in health care workers (LF‐COVID)	Peru	Sep 2020, Nov 2021 (estimated)	SARS‐CoV‐2	To determine the effect of bovine LF on the prevention of COVID‐19 infection	Not yet recruiting

Abbreviations: bLF, bovine lactoferrin; COVID, coronavirus disease; LF, lactoferrin.

^a^ Pre‐print article.

## 4 DISCUSSION

The rapid spread of the SARS‐CoV‐2 virus has brought the scientific community to consider all potential therapeutic agents^54^ and evaluate or re‐evaluate every possible support therapy.^55^ Recently published reviews have studied the role of some micronutrients in support of the immune response against viral infections,^56^ including SARS‐CoV‐2 infection,^57^ but, to the best of our knowledge, no conclusive evidence on LF is available to date. Hence, we systematically collected data on the clinical effects of orally administered LF against viral infections.

In most studies, the glycoprotein was tested in relation to the management of infectious diseases only. This was not unexpected since the eligibility criteria of our systematic review required confirmation of the virus, more easily obtained in chronic conditions. However, a consistent heterogeneity in the findings was observed, both among viral families and within the same family. *Flaviviridae* was the most frequently investigated, and all studies focused on HCV, the major cause of liver disease worldwide, that leads to chronic carriage in 70%–80% of cases with the risk of development of complications such as cirrhosis and cancer.^58^ We found contradictory results in our review in all the considered aspects. Indeed, no clear conclusion could be drawn in relation to LF and either viral, immunological or biological response among HCV patients, even though some weak but positive results were mentioned concerning reduction in viral loads in a few studies. In our opinion, these findings could be mostly attributable to the marked heterogeneity in the recruitment and treatment protocols, especially concerning the LF dose, comparator group and intervention duration. Additionally, most studies were judged of poor quality, highlighting the need to conduct more standardized studies on the topic to reach a final conclusion, even though the recent introduction of pan‐genotypic drugs such as ledipasvir/sofosbuvir and sofosbuvir/velpatasvir^59^ may make these efforts unnecessary. In this regard, it is interesting to note that research on the LF role in HCV infections largely stopped after 2006, probably because of the consistent advancements in treatment effectiveness with the discovery of direct‐acting antivirals that may have caused an interest loss in searching for supplemental treatments.^60^, ^61^

A possible preventive role of LF in the occurrence of confirmed viral infections was evaluated in the child population only. It is well known that human milk, in which LF is naturally abundant,^62^ is of paramount importance in preventing infections and other morbidities in neonates and that its beneficial effects are associated with the volume of intake.^63^ However, in our systematic review an oral supplement of LF did not seem to play a significant role in reducing the incidence of infections sustained by caliciviruses and reoviruses among healthy children.^43^, ^44^, ^50^ Similar findings have been reported in the literature in those studies that quantified the effects of LF on the incidence of gastrointestinal and/or respiratory symptoms without distinguishing the etiological agent^64^, ^65^ (i.e., that did not investigate whether the infections were sustained by viruses or bacteria). By contrast, in the trials selected through our analysis some weak but positive results related to the improvement of the symptoms' duration and severity were observed, in line with the literature in which LF seems to alleviate those symptoms of unspecified microbiological origin more consistently.^30^, ^66^, ^67^

As for HIV, despite the proven efficacy coming from in vitro studies that demonstrated strong inhibition of HIV‐1‐induced cytopathic effect and viral reverse transcriptase exerted by LF,^15^ in our review the oral administration of LF in both children and adults living with HIV was not associated with significant improvements in the disease‐related symptoms or in terms of viral load, although a heterogeneous immune response was described in the children population. These results suggest that LF therapy may have a potential application to help modulating the functions of the immune system,^15^ but further studies are needed to confirm this hypothesis.

Notably, in spite of the scientific interest that arose around LF as a supportive therapy against COVID‐19,^23^, ^68^ we retrieved only two studies that investigated the effects of the glycoprotein on the infections sustained by coronaviruses in clinical settings. Whereas in vitro studies have reported that LF may inhibit coronavirus entry into host cells either by directly binding to the viral particles or blocking the virus receptor or co‐receptor present on the host cell,^23^, ^69^ the evidence we collected in terms of a beneficial effect is limited. However, some encouraging results have been reported,^51^, ^52^ especially in relation to the decrease of symptom severity and duration that, coupled with the optimal tolerance consistently mentioned across the studies, may make the LF supplementation an interesting area for further investigations. Additionally, as a few trials focussing on SARS‐CoV‐2 have just been registered, new data could become available in the near future, allowing a more conclusive judgement on its potential benefits as support therapy.

This study has some strengths and limitations. The main strength is the systematic collection of evidence on the topic. Indeed, to the best of our knowledge, this is the first systematic review on the clinical effects of LF oral administration in the prevention and management of viral infections. We searched both published and unpublished studies, mapping the planned and ongoing research to depict a comprehensive picture of the available data. The limitations to the current review are mostly related to the primary studies included. Heterogeneity in the recruitment and treatment protocols was found, largely limiting the comparability of the results and the opportunity to provide a quantitative synthesis even within the same viral family. In addition, the low methodological quality of the studies included in this review poses a significant challenge in the interpretation of the results. Hence, better‐designed clinical studies (i) using a common pre‐established daily dosage of LF, (ii) for a fixed time period and (iii) in placebo‐controlled homogenous large groups are needed to further study the role of the glycoprotein in the prevention and management of viral infections. Lastly, as confirmation of a viral infection was an inclusion criterion, it is possible that we may not have included a few data on the effects of LF on the infections in which the etiological agent was not specified. However, it was impossible to be sure about the infectious source given the low specificity of the symptoms, and our focus was limited to the glycoprotein's antiviral activity.

To conclude, in vitro studies show evidence in favour of a protective role of LF. However, despite its relatively safe profile, the results from clinical trials investigating LF oral supplementation are still inconsistent, both in preventing and managing these infections. In our opinion, this could be the result of a combination of factors including, but not limited to, small sample sizes, heterogeneity in recruitment and treatment protocols, and low study quality. Hence, further research is needed to better investigate the potential benefits of LF oral administration in relation to viral infections, including SARS‐CoV‐2.

## CONFLICT OF INTEREST

The authors declare no conflict of interest.

## ETHICAL APPROVAL

No ethical approval required for this article.

## AUTHOR CONTRIBUTIONS

Alessandra Sinopoli conceived the study, carried out the literature review, data visualization, analysis and interpretation, and writing‐original draft. Claudia Isonne participated in the data collection, analysis and interpretation, and writing original draft. Maria Mercedes Santoro contributed to data interpretation and manuscript editing. Valentina Baccolini participated in data collection, analysis, interpretation, visualization and manuscript editing. All authors contributed to the article and approved the submitted version.

## Supporting information

Supporting Information 1

## Data Availability

Data sharing is not applicable to this article as no new data were created or analysed in this study.
