# Supplementary material for: The effects of orally administered lactoferrin in the prevention and management of viral infections: A systematic review
Source: Rev Med Virol. 2021 May 28;32(1):e2261. doi: 10.1002/rmv.2261 (PMC9286571; doi:10.1002/rmv.2261)
Supplement: Supplementary file 1 — Supporting Information 1 [file RMV-32-0-s001.docx]

Supplementary Table 1. Search strategies used in the systematic review.

| ("COVID"[All Fields] OR ("coronavirus infections"[MeSH Terms] OR ("coronavirus"[All Fields] AND "infections"[All Fields]) OR "coronavirus infections"[All Fields] OR "mers"[All Fields]) OR "sars"[All Fields] OR ("coronavirus"[MeSH Terms] OR "coronavirus"[All Fields] OR "coronaviruses"[All Fields]) OR "HCOV"[All Fields] OR "HKU1"[All Fields] OR "virology"[MeSH Subheading] OR "virology"[All Fields] OR "viruses"[All Fields] OR "viruses"[MeSH Terms] OR "virus s"[All Fields] OR "viruse"[All Fields] OR "virus"[All Fields]) AND ("lactoferrin"[MeSH Terms] OR "lactoferrin"[All Fields] OR "lactoferrins"[All Fields] OR "lactoferrin s"[All Fields] OR "Lactoferrin") | PubMed |
| --- | --- |
| (lactoferrin AND (covid OR mers OR sars OR coronavirus OR virus OR hcov OR hku1)) | Scopus |
| (lactoferrin AND (covid OR mers OR sars OR coronavirus OR virus OR hcov OR hku1)) | Web of Science |

Supplementary Table 2. Quality assessment of the randomized studies included in the systematic review according to Revised Cochrane risk-of-bias tool for randomized trials (RoB2).

| Author, year | DOMAIN 1 | DOMAIN 2 | | DOMAIN 3 | DOMAIN 4 | DOMAIN 5 | Overall  risk of bias |
| --- | --- | --- | --- | --- | --- | --- | --- |
|  | Risk of bias arising from the randomization process | Risk of bias due to deviations from the intended interventions (effect of assignment to intervention) | Risk of bias due to deviations from the intended interventions (effect of adhering to intervention) | Missing  outcome data | Risk of bias in measurement of the outcome | Risk of bias in selection of the reported result |  |
| Iwasa 2001 | High | High | High | High | Some Concerns | High | High |
| Ishii, 2002 | High | High | High | Some Concerns | Some Concerns | Some Concerns | High |
| Hirashima, 2004 | High | High | High | High | High | High | High |
| Ishibashi, 2005 | High | High | High | High | Some Concerns | Some Concerns | High |
| Kaito, 2006 | High | High | High | Some Concerns | High | Some Concerns | High |
| Ueno, 2006 | Low | Low | Low | Low | Low | Low | Low |
| Yen, 2010 | High | High | High | High | Low | High | High |
| Ochoa, 2013 | Low | Some Concerns | Some Concerns | Low | Low | Low | Some Concerns |
| Sortino, 2019 | Low | Low | Some Concerns | Some Concerns | Some Concerns | Low | Some Concerns |

Supplementary Table 3. Quality assessment of the non-randomized studies included in the systematic review according to the Risk of Bias In Non-randomized Studies of Interventions (ROBINS-I) tool.

| Author, year | Bias due to confounding | Bias in  selection of  participants  into the study | Bias in  classification  of  interventions | Bias due to  deviations from the intended  interventions | Bias due to  missing data | Bias in  measurement  of outcomes | Bias in selection of reported result | Overall  bias |
| --- | --- | --- | --- | --- | --- | --- | --- | --- |
| Tanaka, 1999 | Serious | Serious | Serious | Moderate | Critical | Moderate | Critical | Critical |
| Okada, 2002 | Moderate | Serious | Moderate | Moderate | Moderate | Low | Moderate | Moderate |
| Egashira, 2006 | Serious | Critical | Serious | Serious | Serious | Critical | Serious | Critical |
| Konishi, 2006 | Moderate | Moderate | Moderate | Moderate | Moderate | Low | Low | Moderate |
| Zuccotti, 2006 | Serious | Serious | Moderate | Moderate | Moderate | Moderate | Moderate | Serious |
| Zuccotti, 2007 | Serious | Moderate | Moderate | Moderate | Moderate | Moderate | Serious | Serious |
| El-Ansary, 2017 | Moderate | Serious | Serious | Serious | Moderate | Moderate | Moderate | Serious |
| Serrano, 2020 | Serious | Critical | Serious | Serious | Critical | Critical | Critical | Critical |
| Campione, 2020 | Serious | Serious | Moderate | Moderate | Serious | Moderate | Serious | Serious |
